# Theoretical and experimental revision of surface acoustic waves on the (100) plane of silicon

**DOI:** 10.1038/s41598-021-82211-6

**Published:** 2021-02-02

**Authors:** Alexander Tarasenko, Radim Čtvrtlík, Radim Kudělka

**Affiliations:** 1 FZU-Institute of Physics of the Czech Academy of Sciences, Na Slovance 2, 182 21 Prague 8, Czech Republic; 2 Regional Centre of Advanced Technologies and Materials, Joint Laboratory of Optics of Palacky University, Institute of Physics of Academy of Sciences of the Czech Republic, Faculty of Science, Palacky University, 17. listopadu 12, 77146 Olomouc, Czech Republic; 3 Institute of Physics of the Czech Academy of Sciences, Joint Laboratory of Optics of Palacky University and Institute of Physics AS CR, 17. listopadu 50a, 77207 Olomouc, Czech Republic

**Keywords:** Materials science, Mathematics and computing, Physics

## Abstract

The phase velocity dispersion of the surface acoustic waves on a basal plane of Si(100) has been calculated in the whole range of the azimuthal angle of propagation. We present a detailed description of the calculations. These calculations are compared with the experimental data obtained by a laser acoustic method. Our data convincingly demonstrate the existence of a gap in the spectrum of the phase velocities. The gap means that in a definite range of the phase velocities the SAWs are absent in the whole interval of the azimuthal angles. There is an excellent coincidence between the numerical and experimental data.

## Introduction

There are two types of bulk acoustic waves that can propagate in a homogeneous isotropic elastic solid substrate: the longitudinal bulk wave (LBW) with polarization parallel to the propagation wave vector $\vec{k}$ and transverse (shear) waves with polarization perpendicular to $\vec{k}$. The polarization of the shear waves is either perpendicular or parallel to the surface.

A well-known phenomenon of elastic energy propagation along a free surface of a solid substrate of infinite depth is known as the Rayleigh waves^1^. They propagate with a constant velocity $v_{R}$ close to the shear wave velocity of the substrate $v_{t}$. The energy carried by the Rayleigh wave is concentrated in a one-wavelength-thick wave guide just below the free surface, hence the other popular name—surface acoustic waves (SAWs).

The Rayleigh waves are a mixture of the longitudinal bulk wave (LBW) and the transverse shear bulk wave with the vertical polarization (SVBW), which propagates along the free boundary of solid. They are elliptically polarized in the sagittal plane defined by the propagation vector $\vec{k}$ and normal to the free surface $\vec{n}$. Their phase velocity and group velocity in case of anisotropic media are dependent on the crystallographic orientation. The properties of SAWs are modified on the surface of an anisotropic medium and, in turn, are more difficult for the explicit analytical description. During the past decades the SAWs have been the subject of many studies. We refer the Readers to the excellent books and reviews for the detailed theory of the SAWs^2–6^.

SAWs have attracted considerable experimental interest, because of their employment in many practical applications. Analysis of these waves serves as a useful tool in the nondestructive characterization of materials, especially for the determination of elastic properties of thin films deposited on substrate. SAWs have particular importance in acoustics, seismology, geophysics, telecommunication, material sciences, photonics to name just a few. The SAWs velocities are smaller roughly by five orders of magnitude in comparison to the velocities of the electromagnetic waves in solids, which means 5 orders smaller wavelengths that opens new possibilities in the miniaturization of various devices^7^.

The amplitude of the Rayleigh waves decays exponentially in direction perpendicular to the surface. The penetration depth is of the order of a wavelength and decreases with the increasing of the frequency, which makes the SAWs sensitive to the surface defects, voids, cracks as well as coatings, films, and nano/micro particles adhered to the surface. These surface imperfections cause dispersion of SAWs, i.e. dependence of the phase velocity on the wave frequency. The SAWs are used for the investigations of the solid surfaces, thin films, layers and coatings^8–14^.

Due to the applicational significance of the Rayleigh wave velocity, much effort has been devoted to finding the analytical expressions, which are of simple forms and accurate enough for the practical purposes. There was a need of the reasonably simple even approximate analytical expressions for this quantity. A number of papers devoted to the derivation of the explicit expressions (approximate and exact) for the SAWs velocity had been published and a lot of expressions had been proposed long before the exact solutions. One should mention the first approximate expression for the isotropic material^15^:

1 $$v_{R} = v_{t} \frac{0.87 + 1.12\nu }{{1 + \nu }}, \nu \in \left[ {0 - 0.5} \right]$$

where $v_{t} = \sqrt {E/2\rho \left( {1 + \nu } \right)}$; $\rho$ is the material density; $E$ denotes the Young’s modulus and $\nu$ is the Poisson’s ratio.

Many approximate expressions using sometimes ingenious tricks and approaches have been developed^16–28^. The first exact expression for the root of the Rayleigh equation using Cardano’s method was presented in the work by Rahman^29^. Nkemzi, using the theory of Cauchy integrals, provided an alternate exact expression for the root valid in the whole range of $\nu$^30,31^. However, Malischewsky shown that this solution is incorrect and proposed his own expression using Cardano’s solution^32,33^ (see also Refs.^34–36^).

Despite the fundamental importance, practical relevance and great effort devoted to exploration of elastic surface waves, there is still a need for a complete and general solution for the angular dependence of surface Rayleigh waves for (100) plane of cubic crystals. In this paper we cover the concise introduction to the theory of the SAWs on (100) surfaces of cubic crystals and present all final expressions necessary for calculations of the azimuthal dependencies in the explicit form; the step by step procedure is presented in Supplementary Data. The calculations carried out for the silicon (100) plane were verified by comparison with experimental data obtained by the laser acoustic method.

## Experimental setup

The experimental values of SAWs’ phase velocity have been obtained using the Laser acoustic analyzer (Fraunhofer Institute, Dresden, Germany)^8,9^. The instrument is equipped with a N_2_ pulse laser (wavelength 337 nm) irradiating a sample surface by pulses with duration of 3 ns and energy of 200 µJ. The light is focused to a line of about 4 mm length by a cylindrical lens. This leads to a local thermal expansion and generation of broadband SAWs. The waves are transformed to an electrical signal by a piezoelectric detector using a PVDF foil pressed by a steel wedge to the sample surface (detector receives SAWs within a bandwidth of 200 MHz). The signal is amplified and proceeded to an oscilloscope (sampling rate of 2 GSa/s, bandwidth of 500 MHz) coupled with the PC analyzing the signal. The sample and wedge transducer are fixed to a translation stage driven by the PC controlled DC motor. The stage is moved in the $x_{1}$ direction to vary the distance between the focused line and the transducer. The analysis of the signals detected at different distances permits to calculate the SAWs phase velocity.

Experiments were performed on a one-side polished Si(100) wafer with 4 inches in diameter and thickness of 500 µm. The wafer was 100 Ω·cm phosphorus doped N-type single crystal (University Wafer Inc., US). The phase velocities were measured with a step of 5° in the range [0°–45°], a step of 1° was used for the transition zone around 30°, while 5 measurements were performed for each angle.

The values of phase velocity $\upsilon \left( \theta \right)$ were determined from the dispersion curves $\upsilon \left( {\theta ,\omega } \right)$ by extrapolation to zero frequency. The value $\upsilon \left( {\theta ,0} \right)$ represents the SAWs velocity on the defect-free solid surface. The low frequency SAWs penetrating deep into the crystal effectively eliminate the influence of surface layers and defects^37^. On the clean surfaces the dispersion curves should be straight lines parallel to the frequency axis. In our experiments the dispersion curves $\upsilon \left( \omega \right)$ have very small tilt, indicating the presence of a thin native oxide layer. The estimated thickness of the SiO_2_ layer is about 2–5 nm as evaluated by the same LAW instrument.

The repeatability test on the Si(100) wafer in the [110] direction measured over the distance $x = 20$ mm showed a very low variation of the dispersion curves. Typically, the relative velocity uncertainty was about 0.02%, which corresponds to the absolute uncertainty $\delta v_{R}<$ 1.1 m/s.

## Results and discussion

The anisotropy modifies the structure of the SAW spectrum. There is a number of new “additional” waves, termed as “quasi”, “pseudo” or “leaky” waves and there are some controversies regarding the use of different notations for the same waves. These problems of terminology have been discussed in the review^38^.

### SAWs on Si(100)

Obviously, the spectra of the azimuthal dependencies $\upsilon \left( \theta \right)$ depend significantly on the anisotropy ratio

2 $$A = \frac{{2c_{44} }}{{c_{11} - c_{12} }},$$

where *c*_11_, *c*_12_ and *c*_44_ are the components of the stiffness tensor. In the isotropic cubic materials *A* = 1. For Si *A* = 1.563. This factor determines the azimuthal dispersions of the SAWs. One should expect that the spectra of SAWs calculated for cubic crystals with close values of anisotropy ratio should be qualitatively the same. The azimuthal dispersions have been analyzed in many papers. We mention only some investigations of these dependencies on (100) planes of cubic crystals: Si^39–43^, Cu^44–46^, GaAs^47^, 3C-SiC/c-AlN^48^, InSb^49,50^, Ni^2,51^.

**Figure 1:**
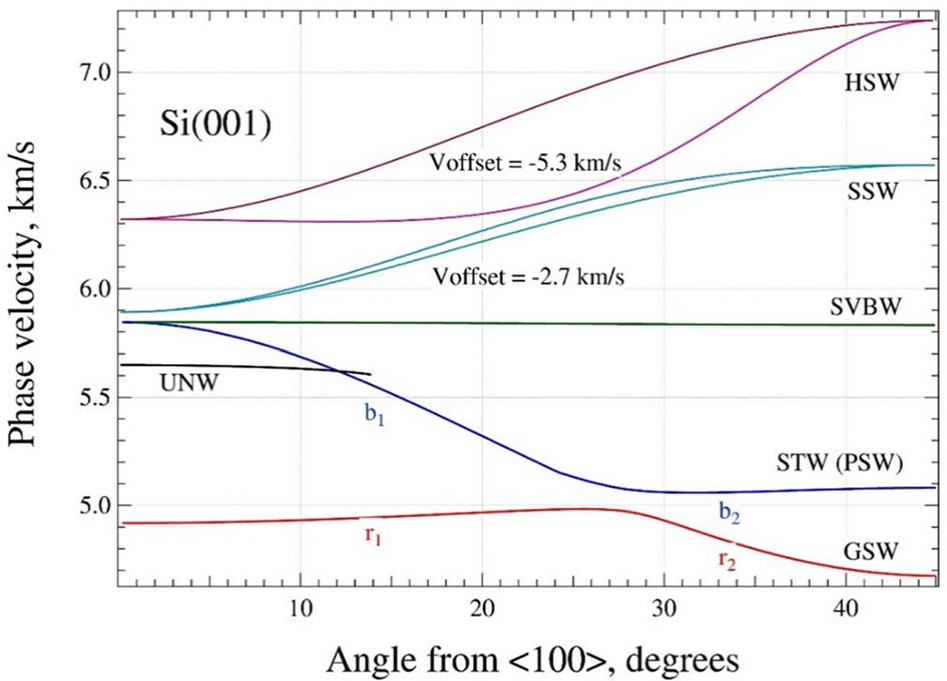
Acoustic wave phase velocities $\nu$ vs $\theta$. (graph created using a software IGOR version 6.34A, https://www.wavemetrics.com/products/igorpro).

Using the approach, presented in the Supplementary Data we calculated these dependencies for the Si(100) plane plotted in Fig. 1. The most interesting are the red and blue lines. These waves appeared in all papers mentioned above, but under the different names and with different understanding as well as interpretation with respect to their layout.

The red line is known as the Rayleigh wave^39,42^ or the generalized surface wave (GSW)^46,48^. It should be noted that the GSWs typically exhibit three displacement components: the longitudinal $u_{l} \left( {\vec{r},x_{3} ,t} \right)$ (along the wave vector $\vec{k}$), transversal $u_{t} \left( {\vec{r},x_{3} ,t} \right)$ (perpendicular to $\vec{k}$ and $\vec{n}$) and normal $u_{3} \left( {\vec{r},x_{3} ,t} \right)$ (along $\vec{n}$). The specific features of GSW are the following:

- the minimal values of the boundary-condition determinant $\delta$ are very small, ∀$\delta \in$ [10^−16^–10^−14^] in the whole range $\theta$, i.e. these values are only the errors of the computations; the wave is a genuine root of the determinant *Det* = 0, contrary to the other SAWs which have the characteristic values $\delta$ ~ 10^–4^–10^–7^;
- the behavior of the displacement components is rather unusual as compared with the other modes.

The blue line is labeled as the slowest (transverse) bulk wave (STW). It is the most controversial mode of the whole spectrum. For the sake of a narrative simplicity, lets divide blue and red curves into parts labeled $b_{1}$, $b_{2}$ and $r_{1}$, $r_{2}$, respectively. The controversy of the angular dispersion of these waves comes from the very often presented composition of STW as a smooth sequence $b_{1}$ and $r_{2}$ parts, while b_2_ part is considered as a disconnected isolated branch or pseudo surface wave (PSW)^39,42,46^. The GSW is treated as the sequence $r_{1}$ and $r_{2}$. Pratt and Lim using a double wedge method (one perspex wedge was fixed and used for the generation of the acoustic signals, while the other one was movable and used for the signal detection) did not reveal a gap in the spectrum^42^. Later, Stoddart et al. using the Brillouin scattering spectroscopy also reported experimental data suggesting the absence of the gap and merging of GSW and PSW branches^40^.

Our results can be treated in a different way. The part $b_{2}$ is not considered as a disconnected branch. It is smoothly connected with the $b_{1}$ part forming the STW (PSW) mode. The GSW is the continuous sequence $r_{1}$ and $r_{2}$. We did not find any solutions in the gap [4.98–5.06] km/s in the whole range of the angles [0°–45°]. Similarly, two distinct well-resolved dispersion dependencies were also obtained for the GSW and PSW modes on GaAs and InSb(001) surfaces^47,49^. It was demonstrated that for the sufficiently high resolution of the Brillouin scattering method it is possible to distinguish experimentally the GSW and PSW modes on GaAs(001). Also, the ultrasonic reflection technique reveals the gap between the GSW and PSW dependencies on Cu(001)^46^.

**Figure 2:**
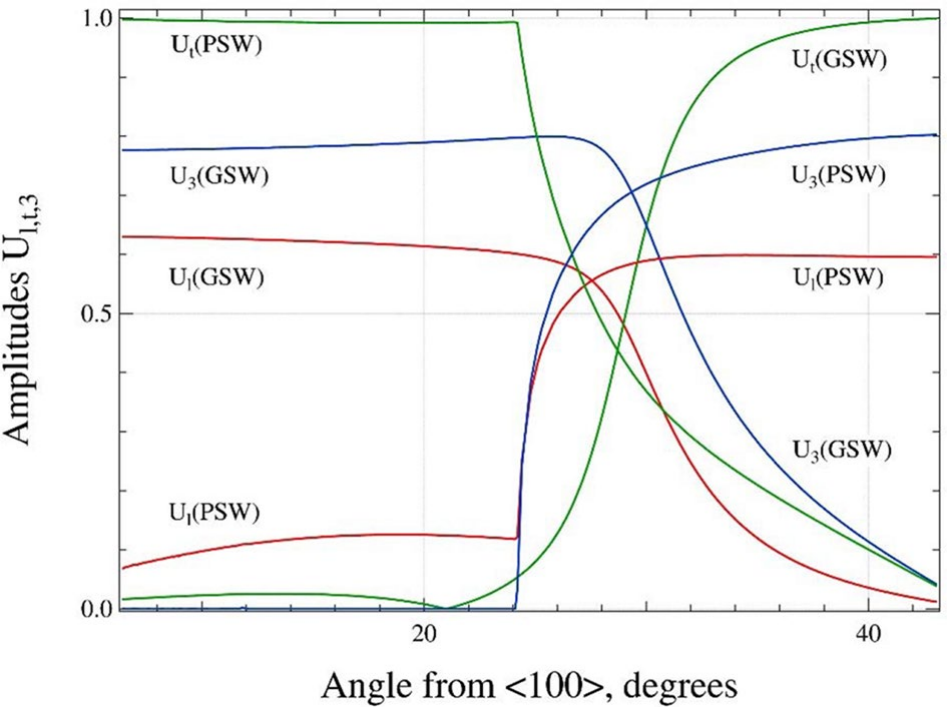
The comparison of the azimuthal dependencies of the normalized longitudinal $U_{l}$ (red), transversal $U_{t}$ (green) and normal $U_{3}$ (blue) amplitudes for the GSW and PSW modes, calculated at $x_{3} = 0$. (graph created using a software IGOR version 6.34A, https://www.wavemetrics.com/products/igorpro).

These modes have distinctly different azimuthal dispersions as compared to the other SAWs. We propose the following possible genesis of these modes based on the analysis of the azimuthal dependencies of their displacement components plotted in Fig. 2. For better representation we calculated the normalized displacements (amplitudes) as

3 $$U_{l,t,3} = \frac{{\left| {u_{l,t,3} } \right|}}{{\sqrt {u_{l}^{2} + u_{t}^{2} + u_{3}^{2} } }}, x_{3} = 0.{ }$$

The dependencies are rather specific. The comparison of the compositions $U_{l} :U_{t} :U_{3}$ of the GSW and PSW modes at the $\theta$ = 0° and $\theta$ = 45°

$${\text{GSW}} \approx \, 0.{6 }: \, 0.0{1 }: \, 0.{8}\quad \theta = \, 0^\circ$$

$${\text{PSW}} \approx \, 0.{6 }: \, 0.0{4 }: \, 0.{8}\quad \theta = { 45}^\circ$$

$${\text{GSW}} \approx \, 0.0{2 }:{ 1 }: \, 0.0{5}\quad \theta = { 45}^\circ$$

$${\text{PSW}} \approx \, 0.0{5 }:{ 1 }: \, 0\quad \theta = \, 0^\circ$$

is surprising and reveals an interesting, obvious symmetry at the ends of the angle range. The behavior of the dependencies is untypical. The GSW amplitudes $U_{l}$ and $U_{3}$ drop down fast for $\theta$ > 27°. The PSW normal amplitude $U_{3}$ is completely absent in the interval [0°–24°]. The $U_{l}$, amplitude is also rather small. The both amplitudes jump in a step-like way at $\theta$ = 24° and the $U_{t}$ amplitude drops down in the same step-like way.

It should be some important reason which causes such an untypical behavior. The possible source of this discrepancy is the coupling between the GSW and STW modes. Without the coupling the modes should have simple monotone azimuthal dispersions: the GSW would be represented by the $r_{1}$-$b_{2}$ sequence and STW behaves as a $b_{1}$-$r_{2}$ sequence, but the “original” modes cross each other at $\theta \approx$ 27°. Having the same velocity, they should interact. The mutual “resonance” interaction results in the instability of the spectrum, prevents the crossing of the modes and produces the reconstruction of the wave modes. In order to accommodate the forbidden crossing, the waves exchange their parts, reconstruct their sequences and form a gap of the forbidden velocities. The result of this reconstruction are new dependencies; $r_{1}$-$r_{2}$ sequence for GSW and $b_{1}$-$b_{2}$ sequence for STW(PSW). It is clearly seen that $b_{2}$ is the Rayleigh-like wave and $r_{2}$ obviously behaves like the STW-type wave.

**Figure 3:**
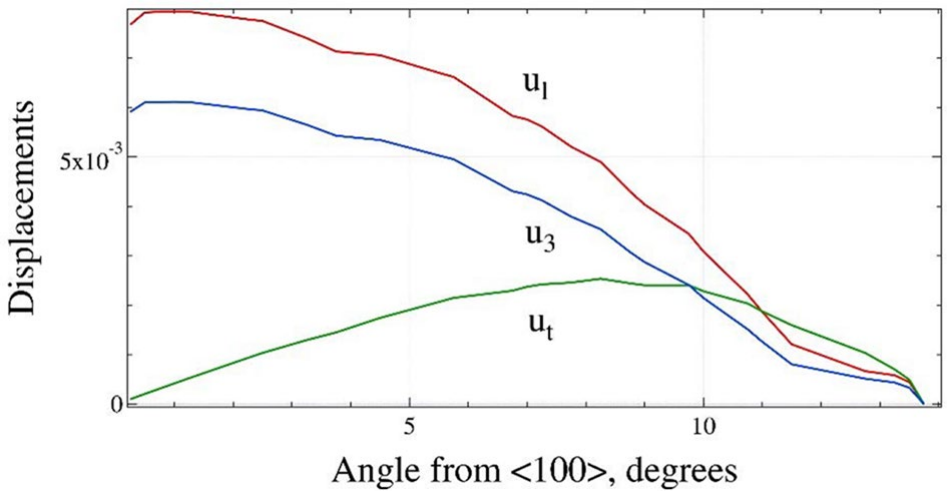
The azimuthal dependencies of the displacements $\left| {u_{l,t,3} } \right|$ for the UNW mode calculated at $x_{3} = 0$ (longitudinal—red, transversal—green and normal—blue). (graph created using a software IGOR version 6.34A, https://www.wavemetrics.com/products/igorpro).

The unknown mode (UNW, black line) exists in a narrow range $0^\circ \le \theta \le 14^\circ$ and has a weak azimuthal dispersion as shown in Fig. 1. For the [100] direction, i.e. $\theta$ = 0°, the longitudinal and normal displacements $u_{l,3}$ have maximal values, while the transversal displacement *u*_t_ has a negligible amplitude. Figure 3 shows that with the increase of $\theta$ the $u_{l,3}$ displacements monotonously decrease. The transversal displacement reaches its maximum for $\theta$ = 8° and further decreases to zero. The compositions $U_{l} :U_{t} :U_{3} \approx 0.3 :0.9 :0.2$ at $\theta$ = 5°changes linearly to 0.6 : 0.6 : 0.47 at $\theta$ = 14°. It should be noted that similar directionally selective/constrained wave considered as shear bulk wave polarized in the sagital plane was also reported by Caliendo^48^ for SiC single crystal.

The almost straight horizontal line (green) represents the shear vertical bulk acoustic wave SVBW polarized in the plane perpendicular to the Si(100) surface regardless of propagation direction^48^. This mode coincides with the STW for [100] direction, which is one of acoustic axes of a silicon single crystal^52^. The azimuthal dispersion is almost absent, but the composition changes noticeably: $U_{l}$ grows from 0.6 to 0.9, $U_{t}$ drops from 0.7 to zero, and only $U_{3}$ ≈ 0.4 has an almost constant value.

The cyan and turquoise lines represent the supersonic waves (SSW, bulk longitudinal wave), or quasi-LBW^2,48^. The “quasi” means that the wave is not exclusively polarized in either parallel or normal to arbitrary propagation direction^38^. The curves are plotted with the vertical offset − 2.7 km/s. These modes have the phase velocity higher than that of the slowest transverse bulk wave. The composition of the waves is almost the same, and almost independent on the $\theta$: $U_{l} :U_{t} :U_{3} \approx 0.9 :0.4 :0.1$. Both waves appear only in the crystals with the strong enough anisotropy of the stiffness tensor. The modes do not satisfy the original condition that the displacement components vanish at the infinite depth. The solution is not a true surface wave. The displacements do not decay with depth to zero, nonetheless, these waves can be quite easily detected. The mode is confidently identified by the search algorithm. SSWs have been observed experimentally using the surface Brillouin scattering^44^.

The highest solutions detected by the search algorithm are two branches of the hypersonic surface waves (HSW, violet and purple lines) with velocities laying in the range [11.6–12.6] km/s. The HSW modes are plotted in Fig. 1 with the vertical offset -5.3 km/s. Both waves have the same composition U_l_ : U_t_ : U_3_ ≈ 0.8 : 0.01 : 0.6 almost independent on the azimuthal angle.

### Experimental data

**Figure 4:**
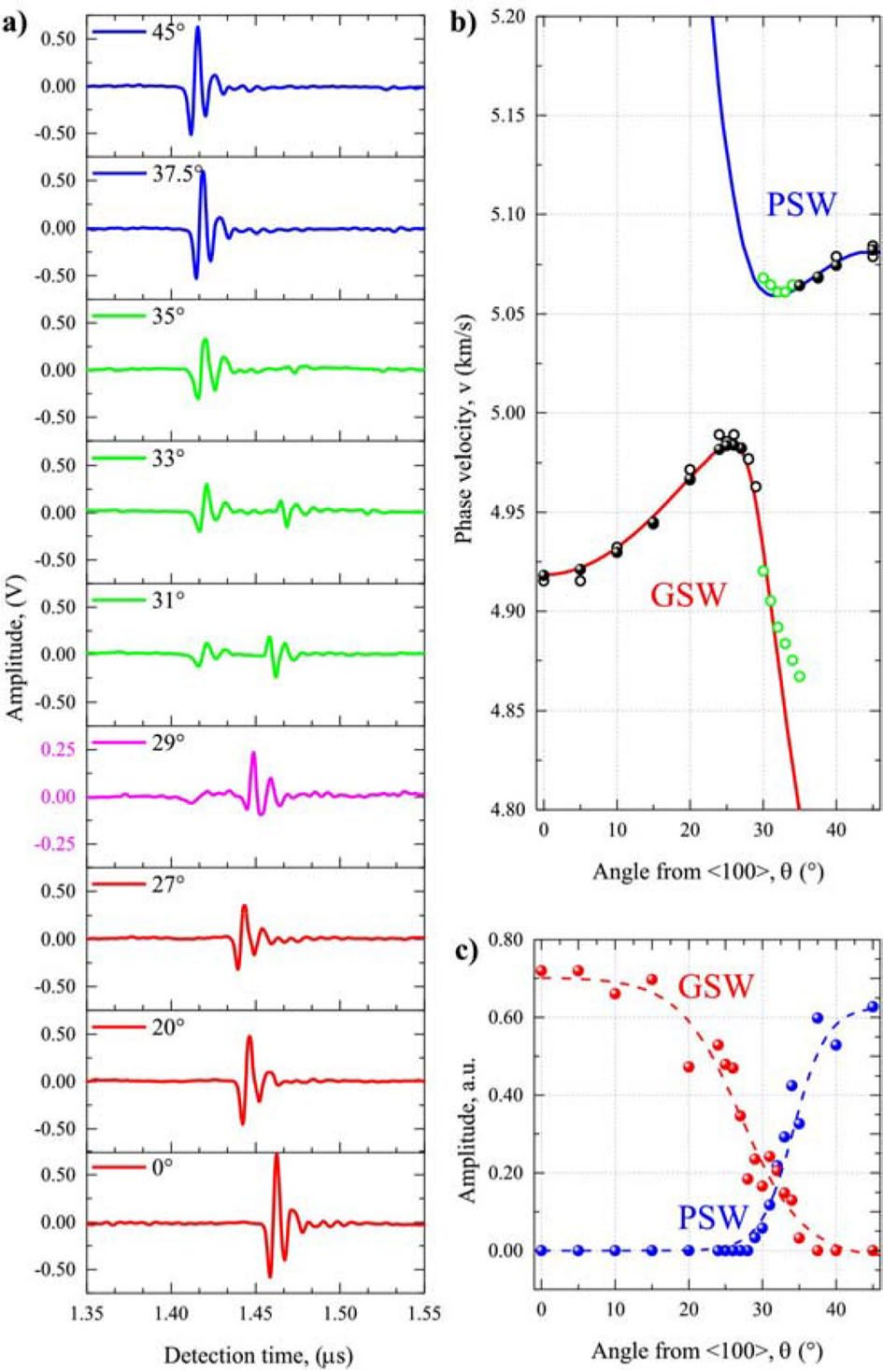
(**a**) The signal waveforms obtained for some representative values of the azimuthal angles *θ*; (**b**) The comparison of the experimental data with the calculated dependencies: solid circles represent data from dispersion curves, open circles represent data from time of detection, green circles highlight the simultaneous detection of GSW and PSW; (**c**) The angular variation of the signal intensities: GSW—red and PSW—blue circles. (graph created using a software OriginPro 8.1 SR3 v8.1.34.90, https://www.originlab.com/).

The experimental values of phase velocities measured via the laser-acoustic method are plotted in Fig. 4b. Due to the symmetry of the (100) plane of the cubic crystals, one needs to measure the SAW angular dispersion only in the 45° interval between the [100] and [110] directions. The propagation vector lies in the (100) plane in the direction specified by abscissa.

The values of phase velocity are depicted as solid circles in Fig. 4b. There is a very good coincidence between the experimental and numerical data, where the experimental values overlap with the theoretical ones. The experimental data follow the GSW branch up to 27°, while they overlap with the PSW branch beyond 35°. In the transition zone around 30° the dispersion curves could not be experimentally measured. On the contrary, the results obtained by Pratt and Lim^42^ and later Stoddart et al*.*^40^ using the Brillouin scattering spectroscopy reported smooth transition between GSW and PSW taking place within this transition zone^40^.

To understand the inability to reconstruct the dispersion curve $v = v\left( \omega \right)$ in the transition region, let us consider the principle of laser-acoustic method, where the dispersion curves $v = v\left( \omega \right)$ are calculated from the Fourier-transformed signals detected at two distances $x_{1}$ and $x_{2}$ (the distance between the laser incidence point and $x_{1}$ is equal 7.2 mm, while the distance $x_{2} - x_{1} = 20$ mm). The typical signal waveforms detected at $x_{1}$ for different wave propagation directions are summarized in Fig. 4a. Except for the angles within [28°–34°] interval, the strong single impulse is observed, which permits the reliable determination of phase velocity from dispersion curves $v = v\left( \omega \right)$. However, for angles in the transition region, either a second impulse appears representing a second mode of waves or the signal waveform exhibits complicated character. In both cases the signals' amplitude and the signal/noise ratio are reduced, especially in the latter case. This phenomenon can be ascribed to the nature of GSW and PSW modes. The dispersion curve cannot be obtained by the standard software.

However, instead of calculating the phase velocity from dispersion curve, it is possible to obtain its value directly from the detection time of the signal at position $x_{1}$ (see open symbols in Fig. 4b). It can be seen that up to the angle of 27° a strong typical waveform can be recognized, while a strong GSW and PSW interaction is evidenced at 29°. On the other hand, two waveforms propagating with different velocities can be recognized already for 30° and can be detected up to 35^°^ (green curves in Fig. 4a) that, in turn, allows determination of two wave velocities (green open circles in Fig. 4b). It can be clearly seen that the measured velocities closely follow the calculated azimuthal dispersion curves. In fact, this is for the first time the LAW method has been successfully employed for simultaneous detection of GSW and PSW.

The origin of the transition region [27°–35°] can be explained based on the variation of the waves with the propagation direction, see Fig. 2. The originally vertical (only for the [100] direction) and later quasi-vertical polarization of GSW starts to twist over to horizontal with the increase of the angle towards [110] due to the coupling with STW^43,52^. The GSW completely degenerates into the STW for the [110] direction. Besides, the penetration depth of GSW increases significantly that in turn leads to the substantial decrease of its normal displacement below the detection threshold for the used LAW setup. This also lowers the detection ability of this wave mode, because only waves with significant polarization component into the sagittal plane can be detected using the LAW experimental setup. Simultaneously, a new high-speed wave mode appears around 30°. This PSW is in fact a leaky-type wave that exists as a superposition of two evanescent partial waves and a small bulk wave component (quasi-shear wave with horizontal polarization). The leaky character represents the loss of energy of PSW away from the surface into the solid while emitting STW as it propagates^43,53^. However, the radiation losses are small and these leaky waves can be easily detected. It should be noted that for the [110] direction the PSW is formed only by two evanescent partial waves as the bulk wave component disappears^44,54^ and turns into the pure Rayleigh wave also classified as the symmetrical supersonic surface wave^38^ and references therein. In fact this transition takes place due to the same reason for which the Rayleigh branch disappeared for this direction^17^.

The dependencies of maximum amplitude of the laser-acoustic impulses on their propagation direction are plotted in Fig. 4c. Based on the absolute value of wave velocity, it is possible to ascribe the individual values either to GSW or PSW. The amplitude of GSW signal decays beyond 15° and vanishes beyond 35°. On the contrary, the amplitude of PSW signal grows within the transition region and reaches its maximum at 45° ([110] direction). The way how the detected laser-acoustic impulses change their propagation velocity, amplitude and shape with the propagation angle implies a smooth transition between two modes, similarly to the Brillouin scattering based methods for the (001) plane of cubic crystals^40,47^. The observed amplitude variations closely reflect the turnover of the polarization of GSW from vertical to transversal direction for GSW (decrease of amplitude) and vanishing contribution of bulk wave component for PSW in the [110] direction (increase of amplitude). Comparison of experimental signal amplitudes detected via a piezo-foil in Fig. 4c and calculated amplitudes presented in Fig. 2 shows a very good qualitative coincidence. This also clearly explains, why GSW and PSW where not detected in the transition region, where their amplitudes were small in order to be experimentally detected using the LAW setup.

## Summary

In this work, we presented a short introduction to the theory of SAWs and all expressions necessary for calculations of the dispersion curves for the SAWs propagating on (100) planes of cubic crystals. We calculated the set of SAWs for the Si(100) plane and obtained some new SAWs. We proposed new interpretation of the genesis of the GSW and STW modes. We demonstrated that the dependencies *δ* vs *θ* may be a useful tool for the analysis of the spectrum of SAWs. We presented very accurate experimental data which exactly coincide with the calculated dependencies. The data clearly prove the existence of the gap in the whole range [0°–45°]. There are no SAWs having phase velocities in the interval [4.98–5.06] km/s for any angle *θ*.

## Supplementary Information

Supplementary Information.

## Data Availability

The data that support the findings of this study are available from the corresponding author upon reasonable request.
