# Supplementary material for: Theoretical and experimental revision of surface acoustic waves on the (100) plane of silicon
Source: Sci Rep. 2021 Feb 2;11:2845. doi: 10.1038/s41598-021-82211-6 (PMC7854651; doi:10.1038/s41598-021-82211-6)
Supplement: Supplementary file 1 — Supplementary Information. [file 41598_2021_82211_MOESM1_ESM.pdf]

Supplementary data

Theoretical and Experimental Revision of Surface Acoustic Waves on the (100) Plane of Silicon

Alexander Tarasenko[1,2], Radim Čtvrtlík[2,3*] and Radim Kudělka[2]

*radim.ctvrtlik@upol.cz

[1]FZU — Institute of Physics of the Czech Academy of Sciences, Na Slovance 2, 182 21, Prague 8, Czech Republic

[2]Regional Centre of Advanced Technologies and Materials, Joint Laboratory of Optics of Palacký University, Institute of Physics of Academy of Sciences of the Czech Republic, Faculty of Science, Palacký University, 17. listopadu 12, 77146, Olomouc, Czech Republic

[3]Institute of Physics of the Czech Academy of Sciences, Joint Laboratory of Optics of Palacký University and Institute of Physics AS CR, 17. listopadu 50a, 77207, Olomouc, Czech Republic

1 Model

1.1 Stiffness tensor

Let $\{x_1, x_2, x_3\}$ be Cartesian coordinates and consider an elastic substrate occupying the half-space $x_3 \leq 0$, with the boundary at $x_3 = 0$. The substrate has the cubic symmetry. Its elastic properties are described by the stiffness tensor c_{ijkl}. Due to the symmetry of the tensor, its indices are collapsed by pairs $11 \to 1, 22 \to 2, 33 \to 3, 23, 32 \to 4, 13, 31 \to 5, 12, 21 \to 6$ (Voigt notation). Cubic crystals have only three independent elastic coefficients:

$$c_{11} = c_{22} = c_{33},$$

$$c_{12} = c_{21} = c_{13} = c_{31} = c_{23} = c_{32},$$

$$c_{44} = c_{55} = c_{66}.$$

The isotropic materials are characterized by only two independent coefficients: c_{11} and c_{12}, but $c_{44} = (c_{11} - c_{12})/2$.

For calculations we use the following values of the coefficients $c_{11} = 165.7, c_{12} = 63.9, c_{44} = 79.56$ GPa (10^9 Pa). The Poisson's ratio and Young's modulus in $\langle 100 \rangle$ direction equal $\nu = c_{12}/(c_{11} + c_{12}) = 0.278$; $E = c_{11} - 2\nu c_{12} = 130.1$ GPa.

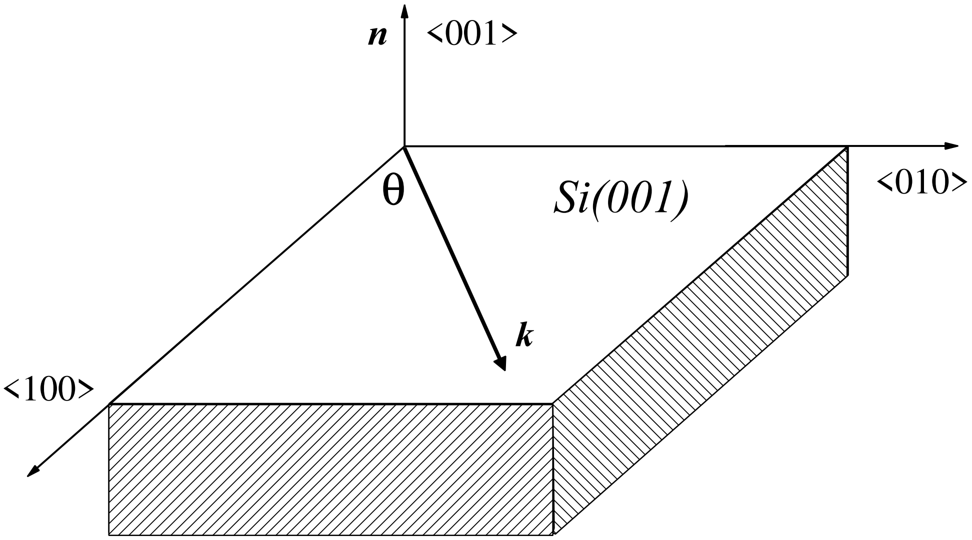

Figure S1: Coordinate system used for the surface-wave calculations.

1.2 Wave equations

The general theory of elastic waves is well developed for the most important cases [1]. A system of the wave equations for the elastic displacements in the substrate crystal should be solved and solutions have to satisfy the boundary conditions for the displacements and stresses at the surface. We mention only the milestones of the theory. The elastic displacements must satisfy the wave equations in the substrate

$$\rho \partial_t^2 u_i - c_{ijkl} \partial_j \partial_k u_l = 0, \quad i, j, k, l = 1, 2, 3. \tag{1}$$

Here, u_i is the component of the displacement $\vec{u}$ along the axis x_i, ρ is the density of the medium and tensor c_{ijkl} is referred to the coordinate system plotted in Fig.S1. Symbols ∂_t and ∂_i denote the partial derivatives $\partial/\partial t$ and $\partial/\partial x_i$, respectively. The summation convention on repeated indices is implied.

We assume the following solutions of the system:

$$\vec{u}(\vec{r}, t) = \vec{\alpha} \exp[\iota \vec{k}\vec{r} + \iota k(a x_3 - vt)], \tag{2}$$

where the propagating vector $\vec{k}$ in the plane $\{x_1, x_2\}$ has components $k_1 = k\cos\theta$ and $k_2 = k\sin\theta$; θ is the angle between $\vec{k}$ and x_1; $\vec{\alpha} = (\xi, \eta, \zeta)$ is a unit (eigen)vector; $v = \omega/k$ denotes the phase velocity; ω is the angular frequency; $\iota \equiv \sqrt{-1}$ stands for the imaginary unit.

They describe plane waves propagating along the direction determined by the wave vector $\vec{k}$. The solution for the displacements can be explicitly determined by substitution of the Eq. (2) into the wave equation (1). It gives the system of Christoffel equations

$$\left(\Gamma - \rho v^2 \mathrm{I}\right) \cdot \vec{\alpha} = 0. \tag{3}$$

Here, Γ and I are the dynamic and identity (unit) 3×3 matrices, respectively. The identity matrix I has entries equal to the Kronecker delta: $\mathrm{I}_{ij} = \delta_{ij}$. The dynamic matrix is symmetrical: $\Gamma_{ij} = \Gamma_{ji}$, which entries have simple forms

$$\begin{aligned}
\Gamma_{11} &= c_{44}a^2 + c_{11}\cos^2\theta + c_{44}\sin^2\theta, \\
\Gamma_{22} &= c_{44}a^2 + c_{44}\cos^2\theta + c_{11}\sin^2\theta, \\
\Gamma_{33} &= c_{11}a^2 + c_{44}, \\
\Gamma_{12} &= (c_{12} + c_{44})\cos\theta\sin\theta, \\
\Gamma_{13} &= (c_{12} + c_{44})a\cos\theta, \\
\Gamma_{23} &= (c_{12} + c_{44})a\sin\theta.
\end{aligned}$$

1.3 Dispersion equation

Eqs. (3) are satisfied nontrivially, provided that

$$\begin{vmatrix}
\Gamma_{11} - \rho v^2 & \Gamma_{12} & \Gamma_{13} \\
\Gamma_{12} & \Gamma_{22} - \rho v^2 & \Gamma_{23} \\
\Gamma_{13} & \Gamma_{23} & \Gamma_{33} - \rho v^2
\end{vmatrix} = 0. \tag{4}$$

The dispersion equation Eq.(4) is a sixth-order algebraic equation in the quantity a [?,?]. It is reduced to the bicubic equation:

$$A_3a^6 + A_2a^4 + A_1a^2 + A_0 = 0, \tag{5}$$

with

$$\begin{aligned}
A_3 &= c_{11}c_{44}^2,\\
A_2 &= c_{44}\left[c_{11}(w_1+w_2) + c_{44}w_3 - (c_{12}+c_{44})^2\right],\\
A_1 &= c_{11}(w_1w_2 - \Gamma_{12}^2) + c_{44}w_3(w_1+w_2) +\\
&+ (c_{12}+c_{44})^2[2(c_{12}+c_{44})\cos^2\theta\sin^2\theta - w_2\cos^2\theta - w_1\sin^2\theta],\\
A_0 &= w_3(w_1w_2 - \Gamma_{12}^2),\\
w_1 &= c_{11}\cos^2\theta + c_{44}\sin^2\theta - \rho v^2,\\
w_2 &= c_{44}\cos^2\theta + c_{11}\sin^2\theta - \rho v^2,\\
w_3 &= c_{44} - \rho v^2.
\end{aligned}$$

The general cubic equation is easily transformed into a cubic equation with a missing quadratic term. We apply the substitution

$$a^2 = z - \frac{A_2}{3A_3}$$

to obtain a simplified, "depressed" equation

$$z^3 + pz + q = 0,$$

where

$$p = \frac{A_1}{A_3} - \frac{A_2^2}{3A_3^2}, \quad q = \frac{A_0}{A_3} + \frac{2A_2^3}{27A_3^3} - \frac{A_1A_2}{3A_3^2}.$$

Let $\epsilon = -(\iota\sqrt{3}+1)/2$ be third root of 1. Then, $\epsilon^2 = (\iota\sqrt{3}-1)/2$, $\epsilon^3 = 1$. Denoting S and T be any values of third roots:

$$S = \sqrt[3]{-\frac{q}{2} - \sqrt{\left(\frac{q}{2}\right)^2 + \left(\frac{p}{3}\right)^3}}, \quad T = \sqrt[3]{-\frac{q}{2} + \sqrt{\left(\frac{q}{2}\right)^2 + \left(\frac{p}{3}\right)^3}}, \tag{6}$$

one can find the three roots z_j of the cubic equation $z^3 + pz + q = 0$ between the nine combinations: $z_j \in \{S\epsilon^l + T\epsilon^m\}$ where $l, m = 1, 2, 3$. There are six roots a_n of the Eq. (5)

$$a_n = \pm\sqrt{z_j - \frac{A_2}{3A_3}}, \quad j = 1, 2, 3, \quad n = 1, 2, \ldots, 6. \tag{7}$$

The locations of these roots have the inversion symmetry. Any partial wave (solution with a definite a_n and corresponding $\vec{\alpha}_n$) satisfies the equations of motion. The general solution is a linear combination of all partial waves

$$\vec{u} = \exp[\iota(\vec{k}\vec{r} - \omega t)] \sum_{n=1}^{6} C_n \vec{\alpha}_n \exp(\iota k a_n x_3). \tag{8}$$

One must choose the non-vanishing values for C_n's, which correspond to the roots laying within the lower half of the complex plane, to ensure decaying of SAWs in the bulk. The imaginary part of the root a_n must be negative: $\Im a_n < 0$. Only three of them satisfy this condition. The rate of decay is chosen as $\Im ak$ in order to simplify formulae. Three constants C_n's are determined from the boundary conditions.

1.4 Eigenvectors

The eigenvectors $\vec{\alpha}_n$ are determined from the homogeneous system of Christoffel equations

$$\begin{aligned}
\left(\Gamma_{11} - \rho v^2\right) \xi_n + \Gamma_{12}\eta_n + \Gamma_{13}\zeta_n &= 0, \\
\Gamma_{12}\xi_n + \left(\Gamma_{22} - \rho v^2\right) \eta_n + \Gamma_{23}\zeta_n &= 0, \\
\Gamma_{13}\xi_n + \Gamma_{23}\eta_n + \left(\Gamma_{33} - \rho v^2\right) \zeta_n &= 0.
\end{aligned} \tag{9}$$

Here, all Γ_{ij} are calculated for a definite value a_n. The component ζ_n is easily eliminated:

$$\Gamma_{23}\left(\Gamma_{11} - \rho v^2\right) \xi_n + \Gamma_{23}\Gamma_{12}\eta_n - \Gamma_{13}\Gamma_{12}\xi_n - \Gamma_{13}\left(\Gamma_{22} - \rho v^2\right) \eta_n = 0.$$

Then,

$$\eta_n = \xi_n \frac{\Gamma_{23}\left(\Gamma_{11} - \rho v^2\right) - \Gamma_{13}\Gamma_{12}}{\Gamma_{13}\left(\Gamma_{22} - \rho v^2\right) - \Gamma_{23}\Gamma_{12}}.$$

Inserting η_n into the 1st equation one gets

$$\zeta_n = \xi_n \frac{\Gamma_{12}^2 - (\Gamma_{11} - \rho v^2)(\Gamma_{22} - \rho v^2)}{\Gamma_{13}(\Gamma_{22} - \rho v^2) - \Gamma_{23}\Gamma_{12}}.$$

ξ_n is determined from the normalization: $\vec{\alpha}_n^2 = \xi_n^2 + \eta_n^2 + \zeta_n^2 = 1$. One should remove the denominator $\Gamma_{13}(\Gamma_{22} - \rho v^2) - \Gamma_{23}\Gamma_{12}$, which for the definite values of the parameters can turn to zero. Let us define

$$D = \left\{\left[\Gamma_{13}(\Gamma_{22} - \rho v^2) - \Gamma_{12}\Gamma_{23}\right]^2 + \left[\Gamma_{23}(\Gamma_{11} - \rho v^2) - \Gamma_{12}\Gamma_{13}\right]^2 + \left[(\Gamma_{11} - \rho v^2)(\Gamma_{22} - \rho v^2) - \Gamma_{12}^2\right]^2\right\}^{\frac{1}{2}}.$$

Then,

$$\xi_n = \left[\Gamma_{13}\left(\Gamma_{22} - \rho v^2\right) - \Gamma_{23}\Gamma_{12}\right] D^{-1},$$
$$\eta_n = \left[\Gamma_{23}\left(\Gamma_{11} - \rho v^2\right) - \Gamma_{13}\Gamma_{12}\right] D^{-1},$$
$$\zeta_n = \left[\Gamma_{12}^2 - \left(\Gamma_{11} - \rho v^2\right)\left(\Gamma_{22} - \rho v^2\right)\right] D^{-1}. \qquad (10)$$

1.5 Boundary conditions

On the free surface all stresses disappear:

$$T_{3j} = c_{3jkl}\partial_l u_k = 0, \text{ at } x_3 = 0 \text{ for } j = 1, 2, 3.$$

It gives a set of three homogeneous equations in the three unknown coefficients C_n. In the explicit form the system is written as follows:

$$\partial_3 u_1 + \partial_1 u_3 = 0,$$
$$\partial_3 u_2 + \partial_2 u_3 = 0,$$
$$\partial_1 u_1 + \partial_2 u_2 + r\partial_3 u_3 = 0, \text{ at } x_3 = 0,$$

where $r = c_{11}/c_{12}$. Using Eq. (8) one gets a system of three homogeneous equations in the three coefficients C_n

$$\sum_{n=1}^{3} C_n(\xi_n a_n + \zeta_n \cos\theta) = 0,$$
$$\sum_{n=1}^{3} C_n(\eta_n a_n + \zeta_n \sin\theta) = 0,$$
$$\sum_{n=1}^{3} C_n(\xi_n \cos\theta + \eta_n \sin\theta + r\zeta_n a_n) = 0.$$

Its determinant must be zero to admit the nontrivial solutions

$$Det = \begin{vmatrix} \xi_1 a_1 + \zeta_1 \cos\theta & \xi_2 a_2 + \zeta_2 \cos\theta & \xi_3 a_3 + \zeta_3 \cos\theta \\ \eta_1 a_1 + \zeta_1 \sin\theta & \eta_2 a_2 + \zeta_2 \sin\theta & \eta_3 a_3 + \zeta_3 \sin\theta \\ \xi_1 \cos\theta + \eta_1 \sin\theta + r\zeta_1 a_1 & \xi_2 \cos\theta + \eta_2 \sin\theta + r\zeta_2 a_2 & \xi_3 \cos\theta + \eta_3 \sin\theta + r\zeta_3 a_3 \end{vmatrix} = 0.$$

The roots determine the velocities of the SAWs. In the simplest case of the isotropic solid the determinant reduces to the well-known Rayleigh equation [?]. In terms of the wave velocities it has the following form

$$\left[2 - (v/v_t)^2\right]^4 = 16\left[1 - (v/v_t)^2\right]\left[1 - (v/v_l)^2\right]. \qquad (11)$$

Here, $v_t = \sqrt{(c_{11} - c_{12})/2\rho}$, $v_l = \sqrt{c_{11}/\rho}$ are the velocities of the transverse and longitudinal bulk waves.

Eq. (11) has a trivial root $v = 0$ and three solutions for v^2. If the Poisson ratio $\nu < 0.263$ there are three real roots. For $\nu > 0.263$, Eq.(11) has one real root and two complex roots. The real root (smallest) gives rise to the Rayleigh wave, which propagates along the surface and decays into the bulk. The other roots, which sometimes are referred as nonphysical, in fact give rise to the leaky surface waves. The full analysis of the roots of the Rayleigh equation was carried out in [**?**].

2 Calculation

The boundary-condition determinant Det is, in general, complex: $Det = \Re Det + \imath \Im Det$. Vanishing of the determinant means the simultaneous zero crossings of the real and imaginary parts. If the real and imaginary parts of the determinant vanish at some value of phase velocity v, it means that the SAW solution exists. The change in sign of the determinant can be used as the search criterion. The code chooses successive values of phase velocity v_0, $v_0 + \Delta$, $v_0 + 2\Delta, \ldots$, until one is found for which the determinant vanishes or changes the sign. The search algorithm, which detects zero crossings, works quite well if Det is real ($\Im Det = 0$), or imaginary ($\Re Det = 0$). But it is not clear if the real and imaginary parts can be simultaneously turned to zero by changing the value of the only one parameter — velocity v. The cases when the absolute value of the determinant is very small but certainly non-zero appear regularly during the computations. The simple and reliable search criterion is the magnitude of the boundary-condition determinant

$$|Det| = \left[(\Re Det)^2 + (\Im Det)^2\right]^{1/2},$$

as the latter provides a single criterion to be examined at the each iteration step of the search algorithm. To get the minimal values of the determinant $\delta = |Det|_{min}$ we use the well-known "golden-section" technique, which reduces the interval $[v, v + \Delta]$ containing δ very fast until the limit of the computer accuracy is reached.

Obviously, the algorithm does not guarantee the value $\delta = 0$. The dependencies of the phase velocity $v(\theta)$ calculated by the "golden-section" algorithm have rather different

values δ. The minimal value δ is infinitely small only for the real roots for the Rayleigh SAWs. In this case δ is the estimation of the accuracy of the calculations. But for the other SAWs this parameter has rather noticeable values. The obvious distinction of this algorithm is a plenty of ("quasi") SAWs discovered by it. The behavior of δ reveals some information about the roots of the determinant. It is interesting that any branch of the obtained waves $v(\theta)$ is characterized by a definite range of δ. For the detailed description of the algorithm we refer the Readers to [2,3].

2.1 Values of the boundary-condition determinant

As it was mentioned above, the SAWs can be classified by the characteristic values of the minimal values of the boundary-condition determinant δ. This is clearly demonstrated in Fig. S2. This feature may be useful sometimes for sorting calculated data in the crossing points or closely placed dependencies.

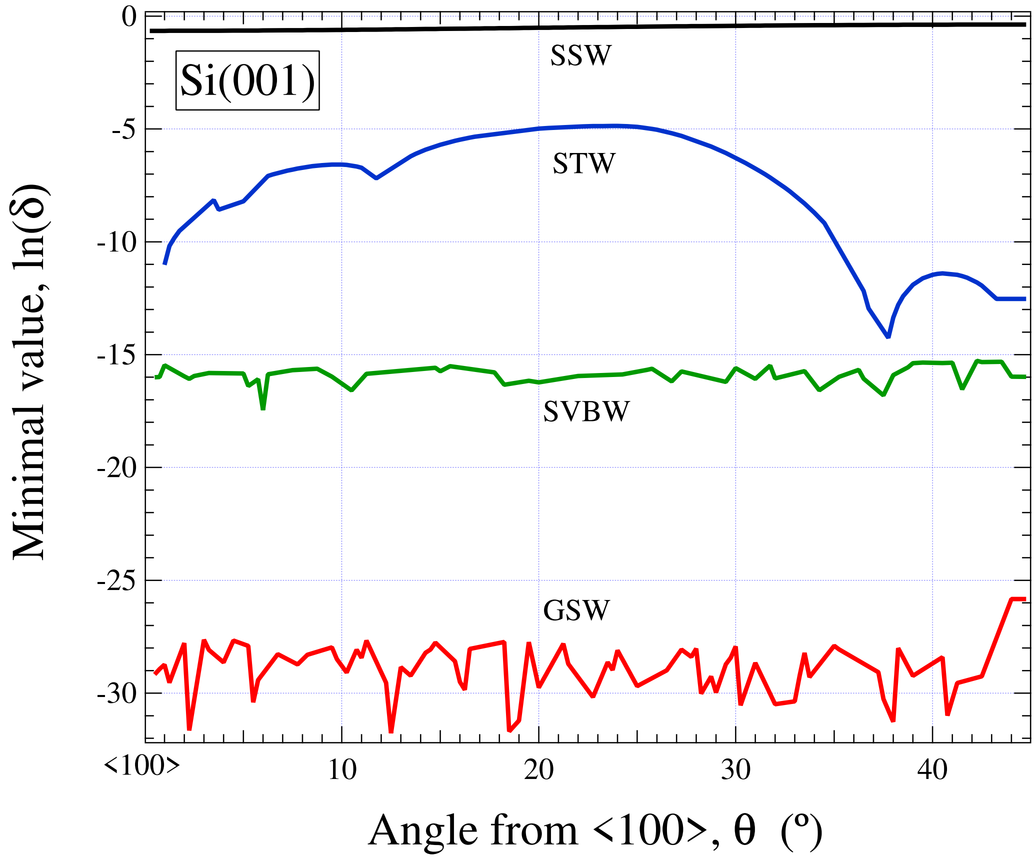

Figure S2: The minimal values of the boundary-condition determinant δ vs. θ for different SAWs.

The GSW mode has the smallest characteristic $\delta < 10^{-12}$. The true value of δ for this mode is zero, of course. The non-zero values arise due to the errors of the calculations. These errors has stochastic origin, which is seen clearly on the plotted dependencies. The SVBW mode has the values δ in an interval $[4 \cdot 10^{-8} \div 3 \cdot 10^{-7}]$. The fluctuations are rather small. The STW mode has very interesting behavior: δ varies in a wide interval $[7 \cdot 10^{-3} \div 3 \cdot 10^{-7}]$ and its behavior is not chaotic, but quite regular. The SSW mode has very large values of $\delta \approx 0.6$. The dependence is a smooth line as the errors of calculations $\sim 10^{-12}$ are invisible.

References

[1] G.W. Farnell, E.L. Adler, in: W.P. Mason, R.N. Thurston (Eds.), Physical Acoustics., v. IX, Acad. Press, NY, 1972, p. 35.

[2] T.C. Lim, and G.W. Farnell, Search for Forbidden Directions of Elastic Surface-Wave Propagation in Anisotropic Crystals. Jour. Appl. Phys. 39 (1968) 4319 – 4325

[3] G.W. Farnell, In: W.P. Mason, R.N. Thurston (Eds.), Physical Acoustics, v. VI, Chap. 3, p. 35, (Acad. Press, NY, 1970)

A Nomenclature

Acronyms

FTW fast transverse bulk wave

GSW generalized surface wave

LAW laser acoustic wave (method)

LBW longitudinal vertical bulk wave

PSW pseudo surface wave

SAW surface acoustic wave

STW slow transverse bulk wave

SHBW shear horizontal bulk wave

SSW supersonic surface wave

SVBW shear vertical bulk wave

Latin letters

a_n roots of the dispersion equation

c_{ijkl} components of the stiffness tensor

c_{11}, c_{12}, c_{44} non-zero components of the stiffness tensor

Det boundary-condition determinant

E Young's modulus

∂_t, ∂_i partial derivatives over t and x_i

I unit matrix

i, j, k, l indices denoting the coordinate axes

$\vec{k}$ wave vector

$\vec{n}$ normal to the surface of the substrate

r ratio of the components c_{11}/c_{12}

t time

T_{ij} components of the stress tensor T

v phase velocity

v_l, v_t the longitudinal and transverse velocities

$\vec{u}$ wave displacement

x_1, x_2, x_3 coordinate axes

z_j roots of the depressed equation

Greek letters

$\vec{\alpha}_n$ eigenvectors

Γ_{ij} components of the dynamic matrix

δ minimal values of the determinant Det

δ_{ij} Kronecker delta

ϵ the third root of 1

λ wavelength

$\iota = \sqrt{-1}$ complex unit

ν Poisson's ratio

ρ substrate density

ξ, η, ζ components of the eigenvector $\vec{\alpha}$

θ angle between the wave vector $\vec{k}$ and x_1 axis

ω angular frequency
